# IL-4 induced MUC4 enhancement in respiratory epithelial cells *in vitro *is mediated through JAK-3 selective signaling

**DOI:** 10.1186/1465-9921-7-39

**Published:** 2006-03-21

**Authors:** Gautam Damera, Baoyun Xia, Goverdhan P Sachdev

**Affiliations:** 1 College of Pharmacy, University of Oklahoma Health Sciences Center, Oklahoma City, OK – 73190, USA; 2 The Oklahoma Center for Medical Glycobiology, University of Oklahoma Health Sciences Center, Oklahoma City, OK – 73104, USA

## Abstract

**Background:**

Recent studies have identified MUC4 mucin as a ligand for activation of ErbB2, a receptor tyrosine kinase that modulates epithelial cell proliferation following epithelial damage in airways of asthmatics. In this study, we investigated the potential role of IL-4, one of the Th2 inflammatory cytokines persistent in asthmatic airways, in regulating MUC4 expression using a cell line NCI-H650.

**Methods:**

Real time PCR analysis was performed to determine concentration and time dependent effects of IL-4 upon *MUC4 *expression. Nuclear run on experiments were carried out to explore potential transcriptional modulation. Western blotting experiments using a monoclonal antibody specific to ASGP-2 domain of MUC4 were performed to analyze MUC4 glycoprotein levels in plasma membrane fractions. To analyze potential signal transduction cascades, IL-4 treated confluent cultures were co-incubated, separately with a pan-JAK inhibitor, a JAK-3 selective inhibitor or a MEK-1, 2 (MAPK) inhibitor at various concentrations before *MUC4 *transcript analysis. Corresponding transcription factor activation was tested by western blotting using a monoclonal p-STAT-6 antibody.

**Results:**

*MUC4 *levels increased in a concentration and time specific fashion reaching peak expression at 2.5 ng/ml and 8 h. Nuclear run on experiments revealed transcriptional enhancement. Corresponding increases in MUC4 glycoprotein levels were observed in plasma membrane fractions. Pan-JAK inhibitor revealed marked reduction in IL-4 stimulated *MUC4 *levels and JAK3 selective inhibitor down-regulated MUC4 mRNA expression in a concentration-dependent fashion. In accordance with the above observations, STAT-6 activation was detected within 5 minutes of IL-4 stimulus. No effect in *MUC4 *levels was observed on using a MAPK inhibitor.

**Conclusion:**

These observations signify a potential role for IL-4 in MUC4 up-regulation in airway epithelia.

## Background

Allergic asthma is an IgE-mediated condition characterized by airway hyper-responsiveness (AHR), chronic airway inflammation and epithelial cell damage [1-3]. These changes in the airways are associated with increased influx of activated CD4+ T-helper (Th) lymphocytes, which in turn, recruit eosinophils via the production of inflammatory mediators, including cytokines (IL-4 and IL-5) and chemokines (eotaxin) [4-7]. The eosinophils upon activation and recruitment cause epithelial cell damage by release of cytotoxic proteins [8-10]. Following tissue damage, the process of epithelial cell proliferation and restitution is broadly attributed to a subclass of receptor tyrosine kinases (RTK) called the ErbB's [11,12]. ErbB family of receptors is composed of four members, namely ErbB1, ErbB2, ErbB3 and ErbB4. Phosphorylation of ErbB receptors by ligand binding induces heterodimerization and activation of specific signaling cascades. The ligands for these receptors are epidermal growth factor (EGF) conserved peptide growth factors [13]. In this context, MUC4, an airway mucin with EGF-like domains in its transmembrane subunit, has been identified as a possible ligand for ErbB2 receptor [14].

MUC4 is a large molecular weight membrane bound O-glycoprotein expressed in the ciliated and goblet cells of the trachea and bronchus [15]. Beyond the respiratory tract, MUC4 is present in the epithelial tissues of stomach, breast, endocervix, cornea and colon [16,17]. Structurally, MUC4 is a heterodimeric complex consisting of a large 850 kD membrane bound MUC4α subunit and a smaller 80 kD trans-membrane MUC4β subunit [18]. The larger MUC4α subunit is believed to exhibit anti-adhesive properties and to protect the apical surfaces of epithelial cells [19]. In contrast, MUC4β subunit possesses two EGF-like domains that bind to ErbB2 receptors and modulates epithelial cell proliferation or differentiation [20]. However, some reports indicate the presence of three EGF domains in the trans-membrane subunit [21].

Clinical and experimental evidence suggests a central role for IL-4 in the development and maintenance of AHR in allergic asthmatics [22]. IL-4 is also reported to play a significant role in secretory cell metaplasia increasing the area of mucus secreting cells in airways. For instance, separate studies with transgenic mice distinctively expressing IL-4 in the lungs showed goblet cell metaplasia [23], allergen challenged STAT-6-deficient mice (IL-4R signaling-impaired mouse airways) showed a marked reduction in the same phenomenon [24]. Furthermore, IL-4 was reported to enhance mucus production in cultured airway epithelial cell line NCI-H292 and to up-regulate *MUC *genes in mouse airways [25].

Earlier, studies involving *MUC *genes were performed to explain a mucus hypersecretory phenotype in chronic airway inflammatory states. Consequently, those studies explored the effects of cytokines and proteolytic enzymes upon a variety of secretory mucin genes including *MUC2*, *MUC5AC*, *MUC5B *and *MUC8*. Findings from these studies revealed a direct effect of inflammatory mediators upon *MUC *gene regulation; nevertheless, ambiguity persists, as to whether the regulatory pattern is exclusive to a few or uniform across all known airway mucin genes. For example, IL-4 decreases *MUC5AC *and increases *MUC8 *levels in cultured human nasal epithelial cells [26]; IL-9 increases *MUC2 *and *MUC5AC *expression and has no effect on *MUC8 *and *MUC5B *genes in bronchial epithelial cells [27]; IL-13 was reported to increase *MUC2 *and decrease *MUC5AC *expression *in-vitro *[28]. Further, the effects of these inflammatory mediators on membrane-bound mucins are not yet defined.

In a previous study, we demonstrated the effects of secretagogues, such as 8-bromocyclic AMP and neutrophil elastase, on mucin secretions using a lung cancer cell line, NCI-H650 [29]. Utilizing the same cell line in the present study, we investigated the effects of IL-4 upon MUC4 gene and glycoprotein expression. Regulation was established to be at the transcriptional level. Using a variety of signaling inhibitors we investigated the activation of janus kinase (JAK) and mitogen-activated protein kinase (MAPK) pathways. We further emphasized the phosphorylation of the related transcription factor, STAT-6.

## Methods

### Cell culture

The human bronchoalveolar carcinoma cell line NCI-H650 (ATCC, Manassas, VA) was cultured in serum free ACL-4 media supplemented with 2 mM glutamine, 100 U/ml penicillin, 100 μg/ml streptomycin and 0.02 mg/ml insulin. Cells were grown at 37°C in CO_2 _fully humidified air and were sub-cultured twice weekly. The cell viability was periodically determined by trypan blue exclusion method.

### Cell stimulation

The confluent cultures, in triplicate, were stimulated with varying concentrations of human recombinant IL-4 (Sigma-Aldrich, Saint Louis, MO). Control groups were treated with media alone. For MUC4 glycoprotein detection, cultures were treated with 2.5 ng/ml of IL-4 for 8 h, washed and re-incubated in fresh medium devoid of IL-4 for an additional 16 h.

Inhibitor studies were carried out by pre-treating cultures separately with 1,4-diamino-2,3-dicyano-1,4-*bis*(2-aminophenylthio) butadiene (U0126), 2-(1,1-dimethylethyl)-9-fluoro-3,6-dihydro-7H-benz [h]-imidazo [4,5-f]isoquinolin-7-one (DBI) and 4-(4'-hydroxyphenyl) amino-6, 7-dimethoxyquinazoline (WHI-P131) in DMSO at varying concentrations (25–100 μM) for 30 min before exposure to IL-4.

### Immunohistochemistry

The presence of IL-4 receptor α chain (part of IL-4R) on the cell surface was established by using a rabbit polyclonal anti-human IL-4Rα antibody (Santa Cruz Biotechnology Inc, Santa Cruz, CA). The harvested cells were initially washed with phosphate buffered saline (PBS) solution, fixed in 4% paraformaldehyde for 5 min and permeabilized in 0.1% Triton X-100. Blocking was performed with 4% BSA for 45 min before incubating with primary anti-human IL-4R α Ab at 1:100 dilutions for 1 h. Secondary incubations were performed with Alexa Fluor^® ^(568 nm) labeled mouse anti-rabbit Ab (Molecular Probes, Eugene, OR) at 1:250 for 10 min. The cells were counterstained with DAPI (Molecular Probes; 0.2 μg/ml in PBS) for 2 min before visualizing on a Zeiss Axioplan 2 microscope. Diluent lacking primary Ab and non-immune rabbit IgG were used as controls.

### RNA extraction and reverse transcription

Total RNA was extracted by RNeasy Mini kit (Qiagen, Valencia, CA) following the manufacture's protocol. The DNase digestion of the RNA samples was performed on RNeasy columns using the RNAse-free DNase set supplied by the same manufacturer. The integrity of the eluted RNA was confirmed by electrophoresing 5 μl of total RNA on 1.2% agarose/formaldehyde gels. The isolated RNA was reverse transcribed using random hexamers and Superscript II First Strand Synthesis kit (Invitrogen, Carlsbad, CA) following the manufacturer's protocol.

### Real-time PCR analysis

Real-time PCR amplifications were performed in the presence of flurogenic *Taq*man 6 Fam-Tamra probes on ABI-Prism 7000 instrument (PE- Applied Biosystems, Foster city, CA). Primers and *Taq*man probes for *MUC4 *were sourced from published reports [30] while the endogenous human 18s rRNA standards were commercially obtained from Applied Biosystems (Foster City, CA) (Table 1). The optimal concentrations for *MUC4 *amplification were determined to be 900 nM of forward, 300 nM of reverse and final probe concentration of 100 nM per reaction. Negative controls were performed omitting the RT step before PCR amplifications. The relative abundance of *MUC4 *was determined by ΔΔCt method.

**Table 1:** Sequence of primers and probes used for Real-time PCR analysis.

Gene	Sequence	Size	GenBank No
*MUC4*	Sense: 5'-GCCCAAGCTACAGTGTGACTCA-3' Antisense: 5'-ATGGTGCCGTTGTAATTTGTTGT-3' Probe: 5'-CGGCCACATCCCCATCTTCTTCAC-3'	102 bp	AF058803
*18S rRNA*	Eukaryotic 18S rRNA Endogenous Controls, Applied Biosystems (Foster City, CA)	186 bp	X03205

### Nuclear run-on transcription assay

The modified assay involving PCR was adopted from earlier published literature by Rolfe, *et al*. [31,32]. Nuclei were extracted from control and IL-4 treated cells after 4 and 8 h using the Nuclei Ez Prep isolation kit (Sigma-Aldrich, Saint Louis, MO). An additional, lyse/wash was included in the protocol to improve the yields of nuclei. Isolated nuclei were layered onto a sucrose cushion (2.1 M sucrose, 5 mM MgAc_2_, 1 mM EDTA, 10 mM Tris-HCl, pH 8, 1 mM EGTA, 1 mM spermidine, 1 mM dithiothreitol and 0.1 mM phenylmethylsulphonyl fluoride) by centrifugation for 40 min at 16000 × *g*. Nuclei from treated and control cells were split into two aliquots. One aliquot (+NTP) was incubated for 45 min at 37°C in a solution containing 20 % glycerol, 30 mM Tris-HCl, pH 8, 150 mM KCl, 2.5 mM MgCl_2_, 1 mM dithiothreitol and 50 U of RNAse OUT^® ^(InVitrogen, Carlsbad, CA) and ATP, CTP, GTP and UTP at 0.5 mM concentration each. The other aliquot (-NTP) was incubated in the same solution without nucleotides. After incubations, RNA was extracted, reverse transcribed and analyzed by real-time PCR as described above.

### Plasma membrane protein extraction

Confluent cultures in triplicate were treated with 2.5 ng/ml of IL-4 or control vehicle alone. The cells were initially washed with ice cold PBS solution and recovered by centrifugation at 600 × g for 5 min. Plasma membrane proteins were isolated and purified by Plasma Membrane Protein Extraction Kit (BioVision, Mountain View, CA), following the manufacturers protocol. Protein content of the purified samples was quantified by BCA assay kit (Pierce biotech, Rockford, IL) using BSA as a standard.

### Western blotting

Equal amounts of protein (50 μg) were resolved separately on 4–20% SDS polyacrylamide gradient gels and transferred to nitrocellulose membranes. The membranes were then blocked by 5% dry milk in Tris-buffered saline (20 mM Tris-HCl, pH 7.2, 150 mM NaCl) for 2 h at room temperature and then incubated with 1:200 diluted human MUC4-specific 1G8 monoclonal antibody (Zymed labs, San Franciso, CA) for 1 h. Secondary antibody incubations were performed with 1:3000 dilution of horseradish peroxidase-conjugated goat anti-mouse IgG antibody. After three successive washes in TTBS (0.5% Tween-20 in Tris-buffered saline), the membranes were treated with HighSignal West Pico chemiluminescent substrate (Pierce biotech, Rockford, IL) and exposed to BioMax films (Eastman Kodak Co, Rochester, NY) for 1 min. Coomassie blue staining of gels was performed to check for variations in sample loading.

For signal transduction experiments, confluent cultures treated with IL-4 for 0, 5, 10, 15 and 20 min were lysed by sonication on ice in lysis buffer (50 mM Tris-HCl, 150 mM NaCl, 1 mM EDTA, 1 mM PMSF, 1% Triton X-100, 0.1% SDS, 1% sodium deoxycholate, 5 μg/ml Aprotinin, 5 μg/ml Leupeptin). Equal amounts of cell lysates (50 μg) were resolved on gels, transferred to membranes and blocked as stated above. Blotting experiments were performed by incubating the membranes overnight in 1:1000 dilutions of human phosphor-STAT-6 mouse monoclonal antibody and human total STAT-6 rabbit polyclonal antibody (Cell Signaling Technology, Beverly, MA). Secondary antibody incubations were performed for 1 h using 1:10000 dilutions of Alexa Fluor 488 goat anti-mouse and Alexa Fluor 532 goat anti-rabbit IgG antibodies (Molecular Probes, Carlsbad, CA). Membranes were washed thrice and scanned using Molecular Imager FX system (Bio-Rad, Hercules, CA) at 488 nm and 532 nm. After Imaging, the blots were stripped and reprobed using human β-actin monoclonal mouse primary antibody (Sigma-Aldrich, Saint Louis, MO) at 1:5000 dilutions.

### Signaling pathway analysis

To understand the signaling mechanism associated with IL-4-mediated *MUC4 *expression, confluent cultures were treated with MAPK-selective inhibitor, U0126, a pan-JAK inhibitor DBI and a JAK3-selective inhibitor, WHI-P131, at 25, 50 and 100 μM concentrations for 30 min. Following inhibitor treatments, the cells were incubated with 2.5 ng/ml of IL-4 for 2 h. Control cultures were treated with DMSO with or without IL-4. After incubations, total RNA was isolated reverse transcribed and analyzed by real-time PCR as described earlier.

### Cytotoxicity evaluation

The evaluation of mediator/inhibitor -influenced cytotoxicity was performed in the above experiments by quantifying the lactate dehydrogenase (LDH) content, using the Cytotoxicity Detection Kit (Roche Diagnostics Corporation, Indianapolis, IN).

### Statistical analysis

Data obtained from all the experiments was analyzed by Kruskal-Wallis one-way non-parametric analysis of variance with post hoc evaluations by Mann-Whitney's rank sum test (SAS Institute, Cary, NC). A level of significance was considered at p < 0.05.

## Results

### IL-4Rα expression in NCI-H650 cells

**Figure 1:**
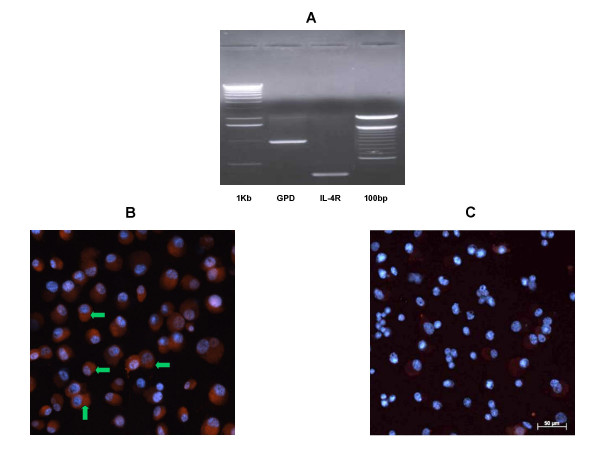
(a) RT-PCR analysis of IL-4R mRNA expression from NCI-H650 cells. Total RNA was extracted from confluent cultures and analyzed for the presence of IL-4R and GAPDH transcripts by RT-PCR. The amplified products were run on 1% agarose-ethidium bromide gels. Bands at 335 bp (IL-4R) and 1000 bp (GAPDH) were observed. Immunohistochemical determination of IL-4Rα upon permeabilized NCI-H650 cells using (b) rabbit polyclonal anti-human IL-4Rα antibody (c) and non-immune rabbit IgG serum (control). Secondary antibody incubations were performed with Alexa Fluor (568 nm)-labeled mouse antibody. Cells were counterstained with DAPI and photographed using dapi: rhodamine filters at 25:2000 ms exposure. Block arrows in (1b) are indicative of specific staining to IL-4 receptors on cell surface.

The expression of IL-4Rα mRNA transcripts was first established by RT-PCR using conditions previously published in the literature [33]. Expected bands at 335 bp for IL-4R and 1000 bp for glyceraldehyde-3-phosphate dehydrogenase (GAPDH) were obtained by running amplified products on 1% agarose-ethidium bromide gels (Fig. 1a).

Localization of IL-4R protein to the cell surface of NCI-H650 cells was established by immunohistochemistry. IL-4R staining was observed on NCI-H650 cell surface using rabbit polyclonal anti-human IL-4Rα antibody but was absent in cells incubated with non-immune rabbit IgG (Fig. 1b, 1c).

### Induction of MUC4 expression by IL-4

**Figure 2:**
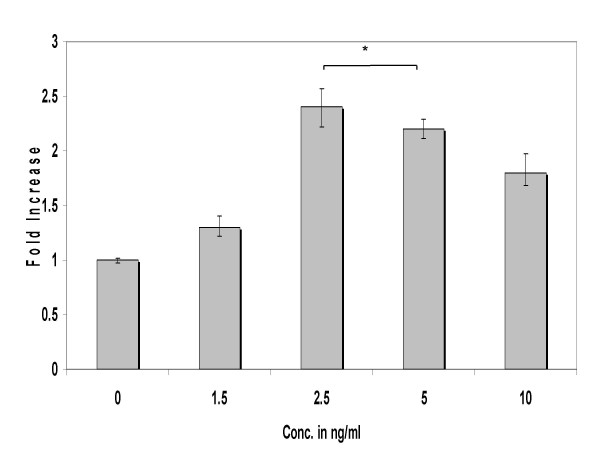
Dose-dependent expression of *MUC4 *by IL-4. NCI-H650 cells were treated with 0 to 10 ng/ml concentrations of IL-4. The mRNA levels of MUC4 were determined by real-time PCR analysis. Data indicated in the graph are mean fold increase ± SE over mean control value. The data are representative of three independent experiments with each treatment run in triplicate. * significantly different, p < 0.05.

To define the effects of IL-4 on steady state MUC4 mRNA levels, confluent cultures were treated with 0 to 10 ng/ml of IL-4 for 2 h. Following treatments, *MUC4 *levels were analyzed by real-time PCR. As shown in Fig. 2, IL-4 up-regulated *MUC4 *expression in a concentration-dependent manner, reaching peak expression at 2.5 ng/ml.

**Figure 3:**
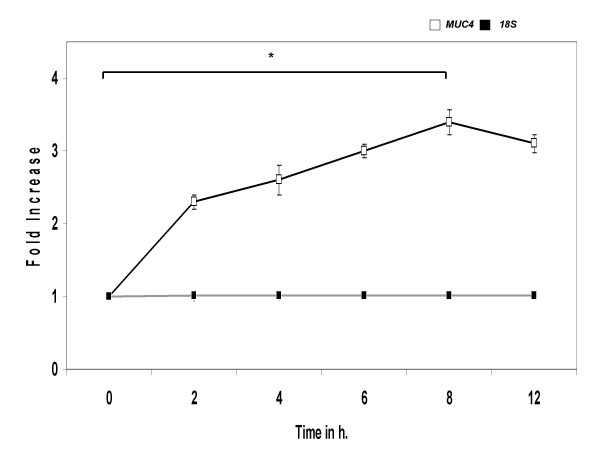
Time course of *MUC4 *gene expression by IL-4. NCI-H650 cells were treated with 2.5 ng/ml of IL-4 for indicated times. Controls were sham treated. [▫] *MUC4 *and [▪] 18S rRNA eukaryotic mRNA levels were determined by real-time PCR amplification. Data indicated in the graph are mean fold increase ± SE over mean control value. The graph summarizes data from three independent experiments with each treatment run in triplicate. * significantly different, p < 0.05.

In order to determine whether IL-4 modulated expression of *MUC4 *is time-dependent, triplicate cultures were incubated with 2.5 ng/ml of IL-4 from 2, 4, 6, 8 and 12 h. MUC4 mRNA levels steadily increased after 1 h and reached maximum expression at 8 h (Fig. 3).

### Transcriptional regulation of MUC4 by IL-4

**Figure 4:**
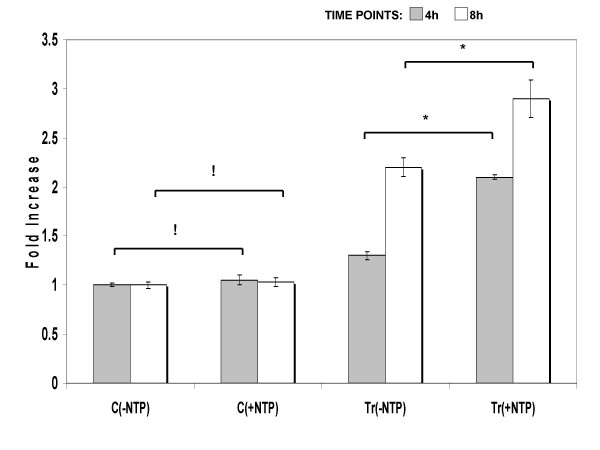
Transcriptional regulation of *MUC4 *by IL-4. Nuclei were isolated from IL-4 treated and control cells at two separate time points of 4 h and 8 h. The extracted nuclei were incubated with or without a mixture of NTP's (0.5 mM each) for 45 min. Real-time PCR amplifications were performed on total RNA extracted from: C(-NTP), untreated nuclei of control cells; C(+NTP), NTP treated nuclei of control cells; Tr(-NTP), untreated nuclei from IL-4-treated cells; and Tr(+NTP), NTP treated nuclei from IL-4-treated cells. Data indicated in the graph are mean fold increase ± SE over mean control value. The graph summarizes data from three independent experiments with triplicate samples. * significant increase (p < 0.05) ; ! no significant difference (p > 005)

To investigate the regulatory mechanism involved in up-regulation of MUC4, nuclear run-on transcription assays were performed. The results revealed, higher *MUC4 *levels in nuclei extracted from IL-4 treated cells incubated with a mixture of NTP's (Tr(+NTPs)) over nuclei from treated cells incubated without NTP's (Tr(-NTPs)). However, no significant difference (p > 0.05) in transcription levels of *MUC4 *were observed between nuclei from control cells incubated with NTP's (C(+NTPs)) over those incubated without NTP's (C(-NTPs)) (Fig. 4).

### MUC4 protein expression

**Figure 5:**
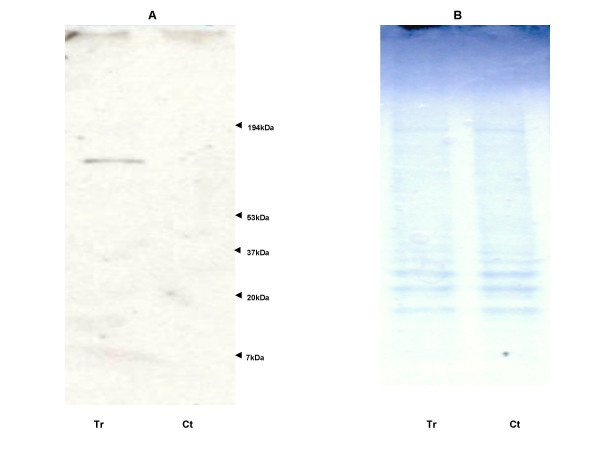
MUC4 glycoprotein expression on IL-4 stimulus. Panel (A): Confluent cells were treated with (Tr) 2.5 ng/ml of IL-4 for 8 h followed by a 16 h chase period in fresh medium without IL-4. (Ct) Untreated control cells. Plasma membrane protein (50 μg) was resolved on 4–20% SDS-PAGE gradient gels and evaluated with 1G8 anti-MUC4 monoclonal primary antibody and horse radish peroxidase conjugated goat secondary antibody. The Western blots are representative of three separate experiments. Panel (B): Commassie blue staining of the gels.

Western blotting experiments using a 1G8 antibody specific to ASGP-2 region of human MUC4 mucin were performed to determine the effects of IL-4 upon MUC4 glycoprotein expression. A specific band at 140 kDa was observed on analyzing the plasma membrane protein preparation in IL-4 stimulated cells (Fig. 5).

### Effects of signaling inhibitors

**Figure 6:**
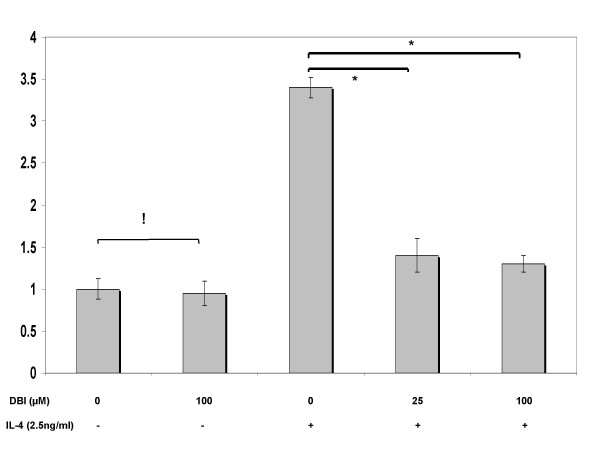
Effects of pan-JAK signaling inhibitor (DBI), upon IL-4-stimulated MUC4 mRNA expression. NCI-H650 cells were pretreated with indicated concentrations of DBI for 30 min before stimulation with 2.5 ng/ml of IL-4. Data indicated in the graph are mean fold increase ± SE over mean control value. The graph summarizes data from three independent experiments with triplicate samples. * significantly different (p < 0.05), ! no statistical significance, (p > 0.05).

**Figure 7:**
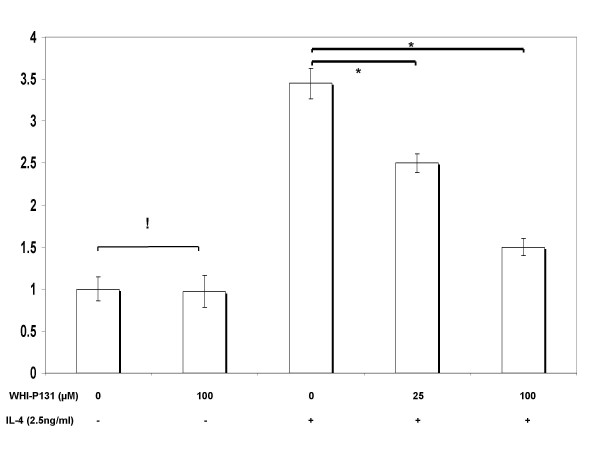
Effects of JAK-3 selective signaling inhibitor (WHI-P131), upon IL-4-stimulated MUC4 mRNA expression. NCI-H650 cells were pretreated with indicated concentrations of WHI-P131 for 30 min before stimulation with 2.5 ng/ml of IL-4. Data indicated in the graph are mean fold increase ± SE over mean control value. The graph summarizes data from three independent experiments with triplicate samples. * significantly different (p < 0.05), ! no statistical significance, (p > 0.05).

**Figure 8:**
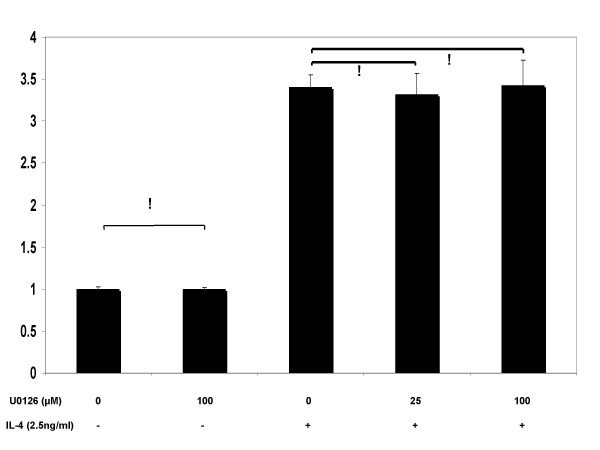
Effects of MAPK signaling inhibitor (U0126), upon IL-4-stimulated MUC4 mRNA expression. NCI-H650 cells were pretreated with indicated concentrations of U0126 for 30 min before stimulation with 2.5 ng/ml of IL-4. Data indicated in the graph are mean fold increase ± SE over mean control value. The graph summarizes data from three independent experiments with triplicate samples. ! no statistical significance, (p > 0.05).

Pre-treatments with signaling inhibitors at 25 μM concentration revealed that the JAK inhibitors DBI and WHI-P131 substantially reduced IL-4-stimulated *MUC4 *expression (Fig. 6, 7). Increasing the concentration of the WHIP131 to 50 (data not shown) and 100 μM, further down-regulated gene expression in a concentration-dependent manner. No significant change (p = 0.4) in *MUC4 *levels was noticed upon increasing DBI concentration from 25 to 100 μM. Cultures pre-treated with U0126 before IL-4 stimulus showed no change (p > 0.05) in *MUC4 *expression over cultures treated with IL-4 alone (Fig. 8).

### STAT-6 activation

**Figure 9:**
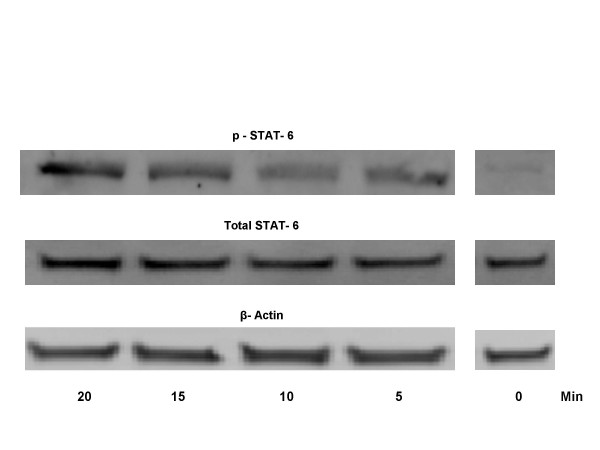
Activation of STAT-6 by IL-4 in NCI-H650 cells. Western analysis of cell lysates from 2.5 ng/ml IL-4 stimulated cells at indicated time points. STAT-6 activation was detected using an anti-phospho-STAT-6 antibody. The Western blots are representative of three separate experiments with triplicate samples.

The activation of STAT-6 by IL-4 is represented by downstream phosphorylation of STAT-6. Therefore, we examined the phosphorylation of STAT-6 (p-STAT-6) in treated and control cells by western blotting using an anti-phospho-STAT6 antibody and anti-STAT-6 antibody. After IL-4 stimulation, elevated p-STAT-6 levels were evident within 5–20 min (Fig. 9). No detectable p-STAT-6 levels were observed in untreated control and IL-4 treated cells at 0 min.

## Discussion

Airway-epithelial cell lines such as A549, Calu-3, HM3, HT29-MTX and H292 have been used as *in-vitro *model systems for *MUC *gene expression studies involving a variety of inflammatory mediators [25,34-37], air pollutants [38] and bacterial endotoxins [39,40]. In an earlier study, a similar cell line, NCI-H650, was demonstrated to secrete mucins in culture conditions by a variety of secretagogues, such as 8-bromocyclic AMP, neutrophil elastase and ionomycin, using a polyclonal antibody to normal human tracheobronchial mucin (HTM-1) [29]. Utilizing the same cell line in the present study, we demonstrated the potential role of IL-4 on membrane bound mucin *MUC4 *regulation in human airways.

The biological actions of IL-4 are initiated by binding to its receptors expressed in varied cell types. Human IL-4R occurs naturally as a membrane bound form and a smaller soluble isoform in airways of asthmatics. The soluble IL-4R lacks the trans-membrane and cytoplasmic domains consistent with the larger membrane bound receptor. Due to the absence of cytoplasmic domains, the soluble receptor upon binding to IL-4 does not induce down-stream signaling cascades [41,42]. In this study, the presence of IL-4R transcripts in NCI-H650 cells was initially determined by RT-PCR experiments. Localization of IL-4R to NCI-H650 cell surface was established by immunohistochemical studies using a rabbit polyclonal antibody specific to the C-terminal cytoplasmic domain of human membrane bound IL-4Rα. Earlier, the presence of membrane bound IL-4R was demonstrated in airway epithelial cells and cell lines [25].

Interestingly, IL-4Rα subunit forms part of the signaling complex for IL-4 and IL-13 receptors [43]. In addition, both IL-4 and IL-13 genes have been reported to be increased 18 h after allergen exposure in patients with allergic asthma [44]. Intranasal instillation of IL-4 or IL-13 in mice developed airway esonophilia and AHR, with no such symptoms in transgenic mice lacking IL-4Rα in airways, further emphasizing the role of IL-4Rα in development of asthmatic phenotype [45]. While emphasizing the critical role of IL-13 in asthma, this study explored the relevance of IL-4 in regulation a membrane bound mucin, MUC4.

Exposure of NCI-H650 cells to IL-4 increased steady state *MUC4 *mRNA in a concentration and time dependent manner, reaching peak expression levels at 2.5 ng/ml and 8 h. Further increasing, the concentration or times of exposure reduced *MUC4 *levels. This phenomenon could be due to release of Suppression of Cytokine Signaling (SOCS) factors that regulate IL-4 mediated gene expression by negative feed back inhibition [46,47]. These results are largely confirmatory of studies where IL-4 was shown to up-regulate *MUC *genes *in-vitro *[25] and *in-vivo *[48]. Our findings stand in contrast to reports where IL-4 down-regulated mucin secretion and up-regulated 15-lipoxygenase enzyme expression (15-LO) in airway epithelial cells [49]. The 15-LO class of dioxygenases enzymes preferentially metabolize exogenous arachidonic acid and linoleic acid to 15-hydroxyeicosatetraenoic acid (15(S)-HETE) and 13-hydroxyoctadecadienoic acid (13-HODE) [50]. The effects of 15-LO metabolites on mucin production are unclear and conflicting reports exist on their ability to regulate mucin production [49,51,52]. Nevertheless, the influence of these mediators in this study would be minimal as we detected an increase in MUC4 mRNA levels within 2 h of IL-4 exposure. Our findings reveal a direct effect of IL-4 upon *MUC4 *gene expression *in vitro *and are based on quantitative PCR methodology.

In this study, transcriptional up-regulation of *MUC4 *was established by nuclear run-on experiments. Our findings are in accordance with previous studies where, transcriptional enhancement of airway *MUC *genes *2 *and *5AC *was demonstrated in response to cytokines, IL-1β [53] and IL-9 [54] respectively, in airway epithelial cells. Conversely, our results differ from reports involving neutrophil elastase (NE), which increased *MUC5AC *and *MUC4 *levels by post-transcriptional mRNA stabilization [37,55]. Interestingly, NE treatment of A549 enhanced *MUC1 *expression at transcriptional level [56]. These reports indicate the regulatory pattern to be both, gene and mediator specific.

Western analysis using a 1G8 monoclonal antibody specific to ASGP-2, a N-glycosylated transmembrane unit of MUC4β, revealed a 140 kDa band in the plasma protein fraction isolated from IL-4-treated NCI-H650 cells. The band obtained was consistent with studies determining MUC4 expression in human corneal epithelium [57], endothelial cells [58] and normal human bronchial epithelial (NHBE) cells following NE exposure [55].

The IL-4 – IL-4R interaction can potentate either JAK or MAPK signaling cascades and consequently, activate STAT-6. Upon activation, STAT-6 dimerizes, translocates to the nucleus, and binds to specific promoter regions to regulate gene transcription [59,60]. With this knowledge, we investigated the potential effects of a pan-JAK inhibitor, DBI, a JAK3-selective inhibitor, WHI-P131, and a MAPK inhibitor, U0126, upon IL-4-mediated *MUC4 *expression. DBI is a potent inhibitor of all members of the JAK family and has been reported to block JAK/STAT-dependent proliferation of CTLL cells following IL-4 stimulus [61]. Alternatively, WHI-P131 is a JAK3-selective inhibitor with no effects on JAK1, JAK2, Syk or Src kinases. WHI-P131 was identified as an anti-thrombotic agent that inhibits JAK3 pathway-dependent platelet aggregation [62]. U0126 is a selective inhibitor of MEK1 and MEK2 with little effect on other kinases such as ERK, PKC, JNK and MEKK. U0126 acts as an immunosuppressant by modulating MAPK dependent IL-2 mRNA levels and blocking T-cell proliferation following antigenic stimulus [63].

In this study, DBI pre-treatments markedly decreased *MUC4 *expression in IL-4 treated cells, however, no change in expression levels were detected between pre-treatments at 25 and 100 μM concentrations. Replication of the experiments with WHI-P131 at 25, 50 (data not shown) and 100 μM concentrations down-regulated IL-4 mediated MUC4 mRNA in a dose dependent fashion. No change in expression levels were detected upon U0126 pre-treatment at varying concentrations with respect to cells treated with IL-4 alone. While, acknowledging the possibility of parallel activation of JAK1 and JAK3 pathways by IL-4, this study explored the significance of JAK3 signaling cascade on MUC4 gene expression. Our results are supportive of earlier reports where JAK3 preferential tyrosine phosphorylation has been reported in response to cytokines that share the common IL-2 receptor γ-chain such as IL-4, IL-7, and IL-9 [64-66]. On the other hand, our results contradict reports where IL-4 treatment has been shown to elevate *MUC2 *levels by a MAPK pathway in human colon cancer cells [67]. These contradictions could, in part be explained by reports, which demonstrated IL-4- dependent MAPK signaling to vary with cell types [60].

Activation of STAT-6 was detected in IL-4 stimulated NCI-H650 cells by western blotting using an antiphospho-STAT-6 antibody. The p-STAT-6 band was evident on resolving lysates from cells incubated with 2.5 ng/ml of IL-4 for 2.5 to 15 min. These findings implicate JAK-mediated STAT-6 activation during IL-4-dependent *MUC4 *enhancement. Our findings are in accordance with studies where another Th2 cytokine IL-9, was reported to activate *MUC5AC *via the JAK/STAT pathway [54].

The molecular mechanisms of *MUC4 *expression have just begun to be elucidated. Recent reports have shown that interferon-γ (IFN-γ) stimulus up-regulates *MUC4 *through enhanced STAT-1 expression in human pancreatic tumor cell line CD18/HPAF. In a similar study, retinoic acid (RA) treatment of the same cells enhanced MUC4 expression through TGF-β2-mediated STAT-1 activation [68]. Simultaneous treatments with RA and IFN-γ showed synergistic induction of MUC4 mRNA. Yet, treatment with RA in this study revealed an inhibition of IFN-γ influenced STAT-1 increase; and exposure to IFN-γ subdued RA influenced TGF-β2 induction. Consequently, the possibility of enhanced *MUC4 *expression through alternate signaling routes during synergistic interaction, distinct from those adopted by their constitutive individual mediators has been hypothesized [69]. In CAPAN-1 and CAPAN-2 cell lines, *MUC4 *promoter activation was influenced by epidermal growth factor (EGF) or transforming growth factor (TGF)-α through a protein kinase C (PKC) cascade [68]. In human esophageal cell line OE33, bile salts transcriptionally regulated *MUC4 *expression via phosphatidylinositol 3-kinase pathway (PI3K) [70].

To date, overall utility of MUC4 to human lung function is unclear; yet, its early expression in human fetal development [71-74] and its specific and timely expression in end-differentiated cell types in adults indicate its potential role in cytodifferentiation [75,76]. Recent studies have identified Muc4 (rat homologue of human MUC4) as a ligand for ErbB2 receptor. The binding of Muc4 to ErbB2 receptor alone or to neuregulin activated ErbB3-ErbB2 heterodimeric complex regulates the expression of p27^kip1^, a cyclin dependent kinase inhibitor. The formation of Muc4-ErbB2 complex up-regulates p27^kip1 ^and promotes cell differentiation, in contrast, Muc4-ErbB2-ErbB3-neuregulin complex formation represses p27^kip1 ^and activates Akt pathways leading to cell proliferation [77]. Further, the ability of SMC/Muc4 to alter ErbB2 localization in polarized human colon carcinoma CACO-2 cells has been demonstrated, indicating a strong physical association between the two molecules [78]. In an elegant study, ErbB2 activation was ascertained for epithelial cell repair following NE exposure [79]. In a similar study, NE treatment significantly enhanced MUC4 (the ligand for ErbB2) in bronchial epithelia cells *in-vitro *[55]. NE is one among a variety of immune cell derived mediators, which modulate airway inflammation and epithelial tissue destruction in chronic respiratory ailments such as CF and asthma.

Numerous studies have hinted at elevated IL-4 expression in bronchoalveolar lavage [80], breath condensate [81] and serum [82] of asthmatics. Further, evaluation of airway biopsies from asthmatic patients has hinted at low, yet increased MUC4 protein levels over normal healthy controls [83]. While acknowledging the important roles of other Th2 cytokines such as IL-5 and IL-13 in regulating *MUC *genes in asthmatic airways, this study explored the relevance of IL-4 upon a membrane bound mucin MUC4 via the common IL-4Rα chain. Our studies revealed that IL-4 induces MUC4 gene and protein levels. The enhancement was determined primarily to be at the transcriptional stage. In addition, inhibitor studies revealed that IL-4 modulates *MUC4 *expression by JAK3 selective-STAT-6 pathway.
